# Egg production patterns of two invertebrate species in rocky subtidal areas under different fishing regimes along the coast of central Chile

**DOI:** 10.1371/journal.pone.0176758

**Published:** 2017-05-08

**Authors:** Marta Blanco, Andres Ospina-Álvarez, Catherine González, Miriam Fernández

**Affiliations:** Núcleo Milenio - Centro de Conservación Marina CCM, Estación Costera de Investigaciones Marinas ECIM, Departamento de Ecología, Facultad de Ciencias Biológicas, Pontificia Universidad Católica de Chile, Santiago, Chile; Northwest Fisheries Science Center, UNITED STATES

## Abstract

Fishing is a major source of human impact, reducing density and size of a wide range of exploited species in comparison to areas exhibiting strong regulations (no-take and partially protected areas, including Territorial Use Rights for Fisheries, TURFs). Since size and density might have important consequences on reproduction, and therefore natural re-seeding, we monitored adult size, density and potential fecundity of the keyhole limpet (*Fissurella latimarginata*) and the red sea urchin (*Loxechinus albus*) in areas under two fishing regimes (TURFs and Open Access Areas, OAAs). Analyzing the distribution of suitable habitats, we predict spatial patterns of potential egg production, to identify reproductive hotspots along the central coast of Chile. The current system of TURFs in central Chile showed higher potential egg production of *F*. *latimarginata* and of *L*. *albus* than expected under a complete OAAs scenario (67 and 52% respectively). Potential egg production showed more than a twofold reduction when the complete TURFs scenario was compared against complete OAAs condition in both species. Individual size and density explained between 60% and 100% of the variability in potential egg production, suggesting the importance of the enhancement of both biological variables in TURFs in Chile. Potential egg production for both species in the northern part of the studied domain was higher due to the combined effect of (a) suitable habitat and (b) concentration of TURFs. Our results suggest that partially protected areas, such as TURFs can significantly enhance the production of propagules that could seed exploited areas.

## Introduction

Coastal zones are among the most impacted ecosystems of the world [1]. Overfishing in particular is one of the most pervasive sources of human impact, that can reduce abundance of target species and propagate effects to the whole community [2–4]. In fact, 63% of assessed stocks and 82% of unassessed stocks currently require rebuilding [5,6]. This situation is of major concern in fisheries that are critically important for diversity and food security, such as artisanal fisheries, which currently concentrate approximately 50% of the world catch and 90% of the fishers worldwide [7]. Therefore, there is an urgent need to improve fisheries management in general and artisanal coastal fisheries in particular. The tools to advance in management of artisanal fisheries range from new ecosystem approaches, total allowable catches as well as spatial management including limited-entry areas (e.g., Territorial Use Rights for Fisheries; TURFs) and permanent fishing bans (e.g., Marine Reserves) [8–10].

The benefits of the permanent fishing bans established in no-take areas have been widely reported, showing higher abundance [11–13], larger adult size of exploited species [14,15]; and also higher species richness [16] in comparison with background areas [17–19]. Partially marine protected areas, including limited-entry TURFs, show in many cases similar patterns than no-take areas in these relevant biological variables (species richness, abundance and adult size of target species) [20, 21], conferring clear benefits over open access or background areas. These results are critically important because very often the implementation of no-take areas generates social resistance [21,22]. Thus, although no-take areas cannot be completely replaced by partially marine protected areas [21], the latter can offer the opportunity of protection enhancing stakeholders’ compromise and secondarily improving our understanding on ecosystems functioning [23–25].

Understanding the performance of fully or partially protected marine areas (no-take areas, TURFs) beyond the limits of the reserve is essential to really show the benefits for fishing grounds recovery and sustainable use of marine resources [19,26–28]. Spill over of exploitable biomass from no-take reserves clearly benefits fisheries [29], and may help gain support for protected areas among stakeholders [30]. Additionally, larger sizes and increased abundances of exploited species inside both fully [18,26] and partially [20] protected areas suggest that either process can enhance reproductive potential in protected areas in general. In fact, empirical evidence predicts between 2 to 5 times higher egg production in protected areas with respect to fished areas, based on a 30% larger commercial size abalone [31]. For snapper (*Pagrus auratus*) relative egg production was estimated to be 18 times higher in no-take reserves than in adjacent fished areas [32]. A 3-times greater gonad production per unit of area was also estimated in mussels inside marine reserves in South Africa, based on the combined effects of increased density and larger size individuals [33]. However, in sites exhibiting larger size individuals inside protected areas, but where density was not amplified, gonad production per unit of area was not higher, suggesting the importance of enhanced density on egg production. Egg production is also expected to change depending on the time elapsed since protection was established, allowing increase in size and abundance of exploited species. In fact, empirical evidence show an annual increase in egg production of lobster (*Jasus edwarsii*) after protection ranging between 4.8% and 9.1% in no-take reserves in New Zealand [34]. However, all these evidences do not account for the direct influence of protection on reproductive investment, as the analyses do not separate the relative importance of the indirect effect of enhancement of size and abundance of adult individuals on reproduction, from the direct influence of protection on gonad investment [35]. Direct estimates of gonad weight in scallops (*Pecten maximus*) suggest that there can be an additional benefit of protection, directly on gonad investment, with gonad weight ranging between 20% and 25% higher in protected areas [36]. Therefore, it is important to assess the relative importance of protection on adult size, density and gonad investment in enhancing reproductive potential of protected areas, since it is essential to understand (a) the influence of fishing effort on reproduction of exploited species, and (b) prospective spillover effects on background areas and networks of marine protected areas. It is also relevant to advance our understanding of the contribution of partially protected areas, such as TURFs that are growing globally, on biological variables beyond diversity, size or abundance.

The coast of central Chile is a good model to examine the influence of relevant, but poorly addressed biological variables, such as egg production, and also the most important variables influenced by human impact (size and density) in determining egg production. First, because the coast of Chile is one of the most productive coastal ecosystems of the world, yet, it is heavily impacted by artisanal fisheries, targeting a large number of fish, invertebrates and algae in rocky shores [37]. Second, because there is a mosaic of human impact, or fishing regimes, that includes few fully protected areas and a system of partially protected areas by fishers (TURFs), interspaced with unregulated fishing zones. Finally, because there is a need to advance in management and conservation plans for coastal areas, identifying the most relevant zones for propagules production [38], and the main determinants. Therefore, the main goal of this study was to develop a spatial model to predict potential egg production along the coast of central Chile, to analyze (a) the influence of human impacts on propagules production; and also (b) the most critical determinant of potential egg production. We chose two economically and ecologically valuable rocky reef species as a model, the keyhole limpet, *Fissurella latimarginata*, and the red sea urchin, *Loxechinus albus* both targeted by artisanal fishers. Thus, our results can have local relevance, but globally can also inform about the most important variables explaining egg production, and the potential losses in reseeding as a consequence of fishing.

## Material and methods

### The study system

The artisanal benthic fishery operating in Chile is characterized by a TURF system that was experimentally established in the early 90s, and formally implemented in the late 90s [37]. Under this TURF system, the fishers are organized within unions that administer a TURF and are obligated to conduct regular stock assessments. Fishers administering a TURF maintain a surveillance system and have exclusive fishing access to that TURF. Each year the fisher unions independently decide on the best local strategy to harvest their assigned quotas, following other nationwide regulations (such as minimum legal size, reproductive bans). However, the majority of the traditional fishing grounds is not under a TURF system and operates as traditional open access areas [20]. In open access areas (OAAs), fishers holding a fishing license can extract benthic resources following national regulations (e.g., minimum legal size, reproductive bans). The fishing licenses restrict fishing activities to regional levels (at the scale of hundreds of kilometers). Minimum legal size and reproductive bans also regulate exploitation of benthic resources in OAAs, but enforcement is poor. Several species are targeted by the artisanal benthic fisheries in central Chile. However, the primary target resources include locos (*Concholepas concholepas*), keyhole limpets (a set of *Fissurella* species) and sea urchins (*Loxechinus albus*) [37].

In spite of the increase in the fraction of the ocean protected in Chile, the coast of central Chile is exhibiting high influence of human impact, and low number (and surface area) of protected areas [37]. For this reason, the co-management system based on Territorial Use Rights for Fisheries (TURF) is seen as critical for management and conservation [39]. First, abundance of benthic resources is higher inside TURFs than in background exploited areas [20]. There is also evidence of larger sized individuals of exploited species inside TURFs [20, 40,41,42]. Moreover, TURFs in central Chile show similar patterns of abundance and adult size of exploited species than no-take areas [20]. Second, social resistance to TURFs is not comparable with no-take areas. In fact, there are only 4 marine protected areas along more than 1000 km of coastline in the most populated region of Chile (from 30°S to 36°S); less than 0.001% protection of the coastal area. In contrast, in the same region there are 97 operative TURFs, covering 30% of the coastal area. Therefore, the TURF’s system confers the opportunity to generate a network of partially protected areas that can serve both, together with other fully protected areas, to sustainable exploitation and to meet conservation goals [20]. For this reason, it becomes important to assess the value of TURFs beyond their limits (e.g., potential egg production) and also, to evaluate what variable enhanced in protected areas (size, abundance, gonad investment) is the most critical determinants of potential egg production.

**Fig 1:**
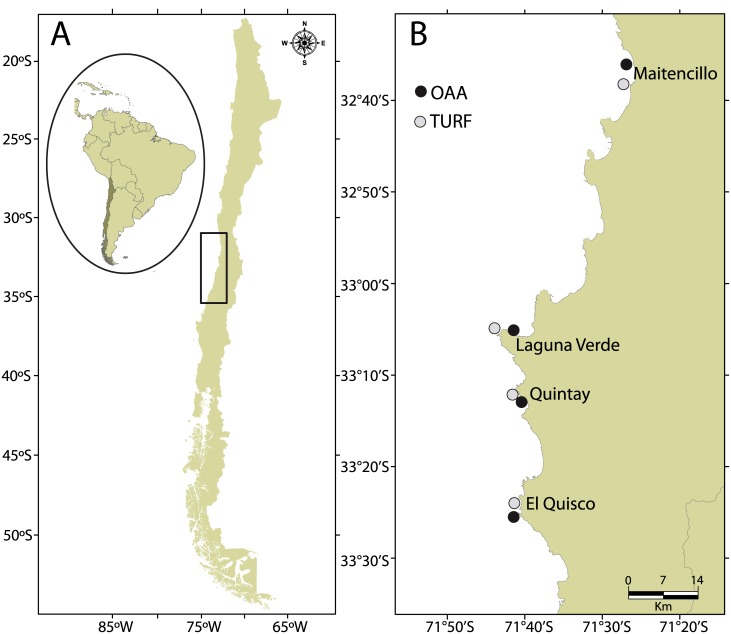
Map of the study area. Maps of Chilean coast showing (A) the study region and (B) the sampling sites where direct collection of organisms was conducted. Fishing regimes are indicated in black circles (Open Access Areas; OAAs) and gray circles (Territorial Use Rights for Fisheries; TURFs).

The predictions of our study cover the coast of central Chile, specifically between 31.57°S and 36.00°S (Fig 1A). In this region there are 75 operative TURFs, covering 31% of coastal area. Along this 795 km of coastline, we selected four sites concentrated between 32.61°S and 33.50°S, were samples were conducted to assess adult size, density and gonad investment. At each site, we selected areas with different fishing regimes: areas limiting fishing access and catches (TURFs) and areas where fishers are not restricted to enter and fish (OAAs). We sampled eight sites, four TURFs and four adjacent OAAs (Fig 1B). At each site, we measured size and density (individuals/m^2^) of both species by direct sampling, during 2012, and estimated potential fecundity (oocytes/female). Using this information, we estimated potential egg production, based on potential fecundity per unit of area per fishing regime.

### Ethics statement

The Chilean Navy and the Undersecretary of Fishing granted all necessary permission and permits to conduct the described fieldwork. Non-destructive manipulation of endangered or protected species was required.

### Estimates of potential fecundity

Potential fecundity, defined as the number of oocytes in the ovary (per female) [41], was estimated. Individuals were collected at the beginning of the reproductive seasons (August—September) reported for *F*. *latimarginata* [42] and for *L*. *albus* [43]. A total of 88 females of keyhole limpets and 75 females of sea urchins, larger than minimum size of reproduction (6 cm and 7 cm, respectively), were collected by professional divers. Specimens were frozen immediately and, later, length and wet gonad weight were recorded for each female. A small weighed fragment (0.2 ± 0.09 mg; mean = 0.119 mg) of the gonad was taken from the ovaries of each female, and preserved in 70% alcohol. Oocytes from each fragment were separated from the ovarian membrane through a washing process [44]. We used a 55 um mesh sieve to collect oocytes, which were then transferred to 100 ml vessel. A minimum of 3 aliquots of 3 ml were used to count the oocytes under the microscope. The number of oocytes was then expressed as the number of oocytes per mg of ovary. Oocytes per female were then calculated extrapolating the count to the total ovary weight. Thus, we were able to estimate potential fecundity, associated to the size distribution of each fishing regime, using the extracted model coefficients (α, intercept) and (β, slope) for the potential fecundity-size relationship described by the following regression model (Eq 1).

$$F=\alpha *\;e^{L\;*\;\beta }$$ (1)

Where F is the potential fecundity (oocytes/female) and L is the size of each individual. Size was measured as peristomial length for keyhole limpets and diameter without spines for sea urchins. Mean potential fecundity per fishing regime was estimated as the average potential fecundity from all individuals of the sample characterizing each fishing regime, for each site.

To estimate potential egg production between fishing regimes, defined as the potential of a given site to produce eggs (oocytes) taking into account density and individual potential fecundity, densities of keyhole limpets and sea urchins were estimated using a visual census. We sampled four 50 m transects per site, positioned perpendicular to the coastline from 3 to 15 m depth, separated 30 m apart from each other. All individuals of both species were counted on a 2 m width strip along transect. We used existing data to determine the fraction of *F*. *latimarginata* from the remaining *Fissurella* species in the sample (see below). To estimate densities per square meter, counts per transect was summed across all stations. Finally, density per square meter was estimated.

In order to characterize size in each fishing regime professional divers took a sample ranging from 29 to 39 keyhole limpets per site (252 keyhole limpets total for fishing regime), and between 17 and 20 sea urchins per site (144 sea urchins total for fishing regime). An exception was El Quisco OAA were only 10 urchins were collected. Total length was measured. Using this sampling we estimated the proportion of females (R) sexing individuals at the laboratory by a dissecting procedure (N = 196 keyhole limpets and N = 144 urchins). We relied on a large sampling conducted in 11 sites in our study region, between 1999 and 2009, to estimate (a) the proportion of *F*. *latimarginata* (K) and (b) the proportion of mature individuals (S). We considered mature individuals those that have reached the minimum size of reproduction (6 cm for *F*. *latimarginata* and 7 cm for *L*. *albus*). Based on size frequency distribution obtained from 9,245 individuals of *F*. *latimarginata* and 9,643 individuals of *L*. *albus*, we obtained the fraction of mature individuals. Based on a sample of 22,456 individuals, we estimated the fraction of *F*. *latimarginata*.

Using Eq 1, we obtained the mean potential fecundity in each site. Density, size, potential fecundity and mean potential egg production were compared between fishing regimes. We considered fishing regime as a fixed factor with two levels TURFs and OAAs. These comparisons were conducted using a Generalized Linear Model (GLM) analysis. For the variable density, we used quasipoisson family error distribution. For the variable potential fecundity and potential egg production we used inverse gaussian and for size we used gaussian family error distribution. The choice of the most appropriate link function and error distribution was made based on residual analyses. We tested the goodness of fitted model with a Chi-Squared test based on residual deviance and degrees of freedom (significance level 0.05). All the statistical analysis were done using R software, version (3.1.3) [45].

### Potential egg production

To determine the spatial variability in potential egg production in relation to fishing regimes, we used the following equations:

$$P_{F.latimarginata}=F*D*R*S*K$$ (2)

$$P_{L.albus}=F*D*R*S$$ (3)

Where P is the potential egg production (oocytes/m^2^), F is the potential fecundity (oocytes/female) from Eq 1, D is the density (individuals/m^2^), R is the proportion of females, S is the proportion of mature individuals and K (parameter used for *F*. *latimarginata* in Eq 2), is the proportion of *F*. *latimarginata* in the sample of keyhole limpets. Parameters R, S and K were assumed fixed while potential fecundity (F) and density (D) varied spatially depending on available rocky shore habitat and fishing regime. Spatial variability in F and D of Eqs 2 and 3, derive from Eqs 4 to 6.

### Spatial data

In order to account for spatial variability of rocky habitat and fishing regimes (TURFs and OAAs) along the coast of central Chile, we developed an indicator of the condition of small fractions of the coast (grids of 2 latitudinal kilometers). Condition was determined based on availability of suitable habitat and fishing regime. First, the coastline was obtained from digitalized Chilean Military Geographic Institute (IGM) charts 1:250,000. Then, we characterized the habitat type using high-resolution satellite photography. Second, we characterized the fishing regime (TURFs and OAAs) based on the presence or absence of TURFs in each coast fragment, using data available from SUBPESCA. All digitalization was conducted with the software ArcGis 9.3. The results were expressed as a percentage of total coast length containing suitable habitat and TURFs in a grid of two latitudinal kilometers. Third, a scaling factor between fishing regime (Ratio TURF/OAA) was estimated to account for differences in density (D) and potential fecundity (F) between fishing regimes (TURFs and OAAs). The factor was calculated for each parameter (D and F) for each species. Finally, we created an indicator to associate the scale of increase in density or potential fecundity (Ratio TURF/OAA) to the percentage of suitable habitat (rocky shores) associated to restricted areas (pTURF). This indicator of spatial variability (Spatial Var) was calculated considering the Ratio TURF/OAA for density (D) and potential fecundity (F), for 134 coastal units (2 latitudinal km grid) along the study area (Eq 4).

Spatial Var=(pTURF*Ratio (TURF/OAA))+(pR−pTURF) (4)

Where pR is the percentage of coastline with rocky shore habitat. In order to obtain the indicator of spatial variability (Spatial Var) for each coastal unit, a linear regression between the indicator (Spatial Var) and the variables of interest (potential fecundity and density) was estimated for the 8 grids containing the areas sampled in our study. Then, we used these relationships to predict potential fecundity (F) and density (D) for the 134 units of our regional domain.

$$D=Spatial\;Var*\beta_{D}$$ (5)

$$F=Spatial\;Var*\beta_{F}$$ (6)

Where *Spatial Var* is the value obtained by Eq 4, β_D_ is the slope of the regression between density (D) and Spatial Var, and β_F_ is the slope of the regression between potential fecundity (F) and Spatial Var. When Spatial Var is 0, the value of density, and therefore potential fecundity, is always 0 (not suitable rocky habitat). Using all these estimates, we were able to calculate potential egg production (P) for the study area:

$$P_{F.latimarginata}=\left(Spatial\;Var*\beta_{F}\right)*\left(Spatial\;Var*\beta_{D}\right)*R*S*K$$ (7)

$$P_{L.albus}=\left(Spatial\;Var*\beta_{F}\right)*\left(Spatial\;Var*\beta_{D}\right)*R*S$$ (8)

In order to determine the influence of available rocky habitat and fishing regime on predicted potential egg production, we compared the predicted potential egg production (dependent variable), in 134 coastal units, across the values of percentage of coastline with rocky habitat (pR) and the percentage of coastline with rocky habitat associated to TURFs areas (pTURF) (both as independent variables). These comparisons were conducted using a GLM analysis with a quasipoisson family error distribution. Furthermore, to evaluate the influence of human impacts on propagules production we compared two scenarios: (a) a full restricted access scenario against a full open access and (b) the current system of TURFs against a full open access scenario.

In order to define the most critical determinant of potential egg production, we compared the mean egg production per site (dependent variable), across mean density and mean size (independent variables) obtained in each site using an ANCOVA analysis.

## Results

### Density and potential fecundity

The relationship between potential fecundity (F) and total length (L) was described by an exponential model function for both species, showing the expected increase of potential fecundity with size. Estimated potential fecundity was F = 548,500 (258,100) e^L^*^0.31 (±0.05)^ (df = 87, p-value < 0.01) for keyhole limpet and F = 1,745 (1,338) e^L^*^0.74 (±0.08)^ (df = 75, p-value < 0.01) for sea urchin.

**Fig 2:**
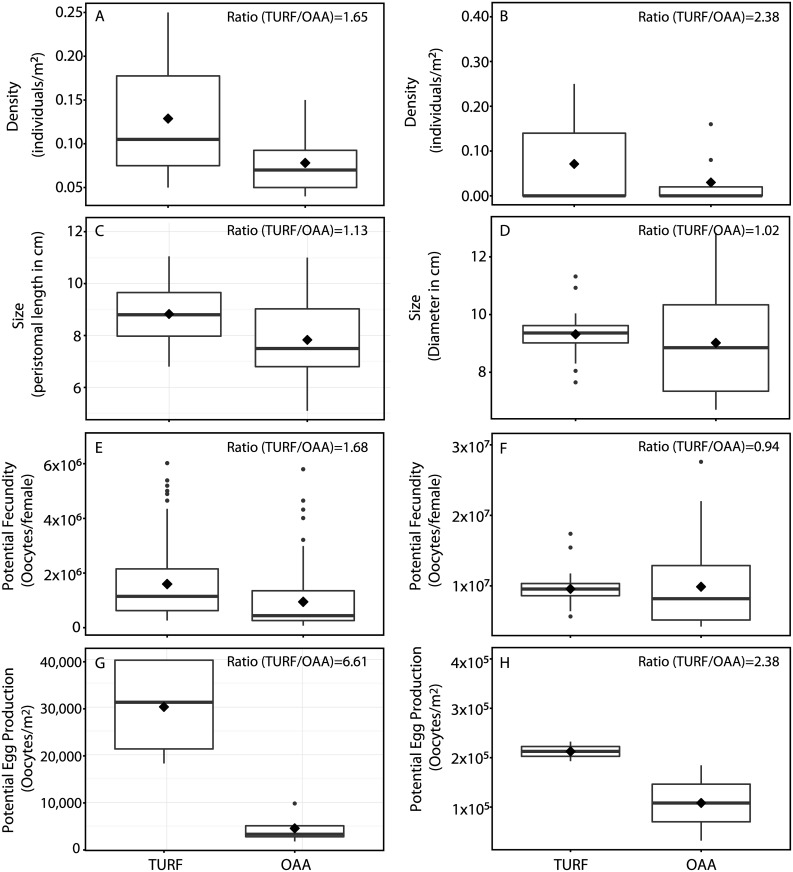
Biological variables between fishing regimes. Box plots showing density, size, potential fecundity and potential egg production in areas under different fishing regimes: Territorial Use Rights for Fisheries (TURF) and Open Access Areas (OAAs; not limiting the entry of fishers). The number in the right corner in each plot indicates the ratio between fishing regimes (TURF/OAA) for each variable. Black diamonds’ indicate mean: (A) *Fissurella latimarginata* density; (B) *Loxechinus albus* density; (C) *F*. *latimarginata* size; (D) *L*. *albus* size; (E) *F*. *latimarginata* potential fecundity; (F) *L*. *albus* potential fecundity; (G) *F*. *latimarginata* potential egg production; and (H) *L*. *albus* potential egg production.

Fishing regime showed a significant effect on mean size (L), density (D), potential fecundity (F) and potential egg production (P) of *F*. *latimarginata* (p-value always < 0.05) (Table 1A). On average, density of keyhole limpets was 65% higher in TURFs compared with OAAs (Fig 2A). Size was also significantly different between fishing regimes, although the change was smaller (13%; Fig 2C). Potential fecundity was also 68% greater in TURFs in comparison to OAAs. Similarly, potential egg production was six-fold higher in TURFs than in OAAs (Fig 2E and 2G, Table 1). In contrast, no significant effect of fishing regime on density, size, potential fecundity and potential egg production was observed for *L*. *albus* (Table 1B). Although density of sea urchins in TURFs appeared to be two-fold higher than in OAAs, the large variability observed among sites in this species did not yield statistical differences (Fig 2B). Sea urchin size was not significantly different between fishing regimes (Fig 2D, Table 1B). No difference in potential fecundity and high variability were also observed between TURFs and OAAs (Fig 2F, Table 1). Similarly, the influence of fishing regime on potential egg production was not significant despite an apparent two-fold larger potential egg production in TURFs (Fig 2H, Table 1).

**Table 1:** Generalized linear models. Statistical results of the generalized linear models (GLM) applied to density, individual size, potential fecundity and potential egg production, across two fishing regime: TURFs (Territorial Use Rights for Fisheries) and OAAs (Open Access Areas).

**A. *Fissurella Latimarginata***					
Model: glm (Density ~ Fishing Regime, family = quasipoisson)					
Deviance Explained: 18.47%					
	Df	Deviance	Resid. Df	Resid. Dev.	Pr (>Chi)
NULL			31	0.95	
Fishing Regime	1	0.20	30	0.75	0.01
Model: glm (Size ~ Fishing Regime, family = gaussian)					
Deviance Explained: 14.20%					
	Df	Deviance	Resid. Df	Resid. Dev.	Pr (>Chi)
NULL			251	440.99	
Fishing Regime	1	62.64	250	378.36	1.25*10^−10^
Model: glm (Potential Fecundity ~ Fishing Regime, family = inverse.gaussian)					
Deviance Explained: 4.82%					
	Df	Deviance	Resid. Df	Resid. Dev.	Pr (>Chi)
NULL			251	2.87*10^−4^	
Fishing Regime	1	1.84*10^−5^	250	2.73*10^−4^	7.90*10^−5^
Model: glm (Potential Egg Production ~ Fishing Regime, family = inverse.gaussian)					
Deviance Explained: 57.71%					
	Df	Deviance	Resid. Df	Resid. Dev.	Pr (>Chi)
NULL			7	9.43*10^−4^	
Fishing Regime	1	5.44*10^−4^	6	3.98*10^−4^	0.005
**B. *Loxechinus albus***					
Model: glm (Density ~ Fishing Regime, family = quasipoisson)					
Deviance Explained: 6.93%					
	Df	Deviance	Resid. Df	Resid. Dev.	Pr (>Chi)
NULL			15	1.99	
Fishing Regime	1	0.13	14	1.86	0.31
Model: glm (Size ~ Fishing Regime, family = gaussian)					
Deviance Explained: 1.47%					
	Df	Deviance	Resid. Df	Resid. Dev.	Pr (>Chi)
NULL			143	214.90	
Fishing Regime	1	3.17	142	211.73	0.14
Model: glm (Potential Fecundity ~ Fishing Regime, family = inverse.gaussian)					
Deviance Explained: 0.17%					
	Df	Deviance	Resid. Df	Resid. Dev.	Pr (>Chi)
NULL			143	2.22*10^−6^	
Fishing Regime	1	3.64*10^−9^	142	6.21*10^−6^	0.63
Model: glm (Potential Egg Production ~ Fishing Regime, family = inverse.gaussian)					
Deviance Explained: 13.87%					
	Df	Deviance	Resid. Df	Resid. Dev.	Pr (>Chi)
NULL			3	2.13*10^−5^	
Fishing Regime	1	2.96*10^−6^	2	1.83*10^−5^	0.43

### Spatial variability in potential egg production

The spawning fraction (R) for *F*. *latimarginata* was 0.48 and for *L*. *albus* was 0.44. The fraction of mature individuals (S) was 0.84 for *F*. *latimarginata* and 0.69 for *L*. *albus*. The fraction of *F*. *latimarginata* in the keyhole limpet samples (K) was 0.43 (S1 Table). These estimates were used as constants in Eqs 2 and 3. The linear models selected to describe the relationship between density (D) and potential fecundity (F) against the indicator Spatial Var were statistically significant (Table 2).

**Table 2:** Linear regressions. Linear models relating density and potential fecundity, both dependent variables and Indicator (Spatial Var; independent variable). Linear regressions were used to obtain the relationship between the indicator (Spatial Var) and the variables studied (potential fecundity and density).

***A*. *Fissurella latimarginata***				
Model: lm (Density ~ Spatial Var- 1)				
R^2^ = 0.86				
	Estimate	Std. Error	T value	Pr (>| t |)
Slope (β_D_)	1.08*10^−3^	1.52*10^−4^	7.17	1.8*10^−4^
Model: lm (Potential Fecundity ~ Spatial Var—1)				
R^2^ = 0.56				
	Estimate	Std. Error	T value	Pr (>| t |)
Slope (β_F_)	11977	3592	3.33	0.01
*B*. ***Loxechinus albus***				
Model: lm (Density ~ Spatial Var—1)				
R^2^ = 0.87				
	Estimate	Std. Error	T value	Pr (>| t |)
Slope (β_D_)	3.52*10^−4^	6.53*10^−5^	5.39	0.01
Model: lm (Potential Fecundity ~ Spatial Var—1)				
R^2^ = 0.88				
	Estimate	Std. Error	T value	Pr (>| t |)
Slope (β_F_)	123780	15395	8.04	8.83*10^−5^

Using the coefficients estimated by the linear regressions in the study sites, P (potential egg production) was predicted for the 134 units in the regional domain of our study as follows:

$$P_{F.latimarginata}=\left(Spatial\;Var*11,977\right)*\left(Spatial\;Var*0.00108\right)*0.48*0.84*0.43$$ (9)

$$P_{L.albus}=\left(Spatial\;Var*123,780\right)*\left(Spatial\;Var*0.00035\right)*0.44*0.69$$ (10)

**Fig 3:**
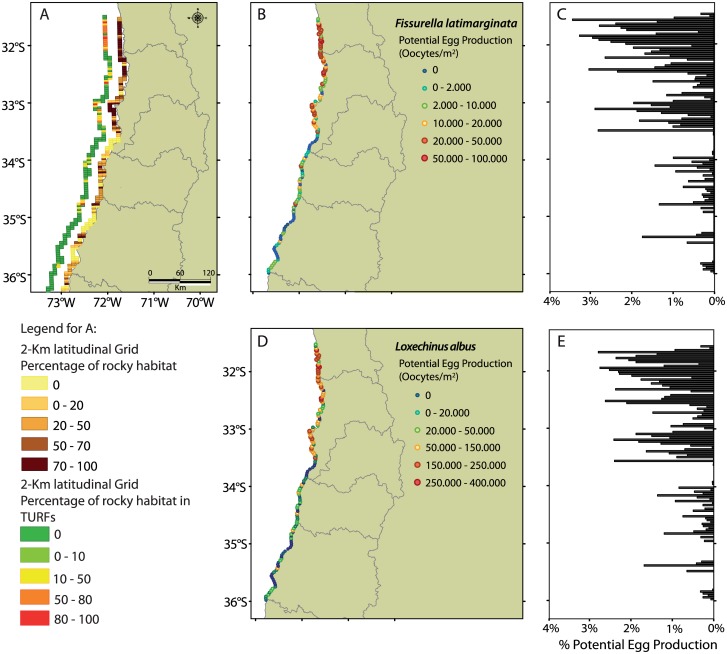
Spatial distribution of habitat, restricted access areas and potential egg production. Spatial maps showing (A) percentage of coastline with rocky habitat and restricted access (Territorial Use Rights for Fisheries, TURFs) and (B, D) predicted potential egg production (oocytes/m^2^) along a latitudinal gradient with a 2-km grid resolution. Plots C and E show the contribution (percentage) of oocytes/m^2^ (potential egg production) of each grid to the regional (study area) production.

In the study zone, 379 of 795 km of coastline (47.7%) between 31.57°S and 36.00°S showed rocky habitat suitable for each species under study. We estimated that 21.27% (169 km) of coastline exhibited restricted access, distributed in 75 operative TURFs. The highest percentage of rocky habitat under TURF was found between 31.57°S and 33.50°S (49.94% of the coastline), while in the southern section of the study region (33.50°S to 36.00°S) fewer TURFs associated to suitable rocky habitat were present (27.79% of the coastline; Fig 3A).

**Fig 4:**
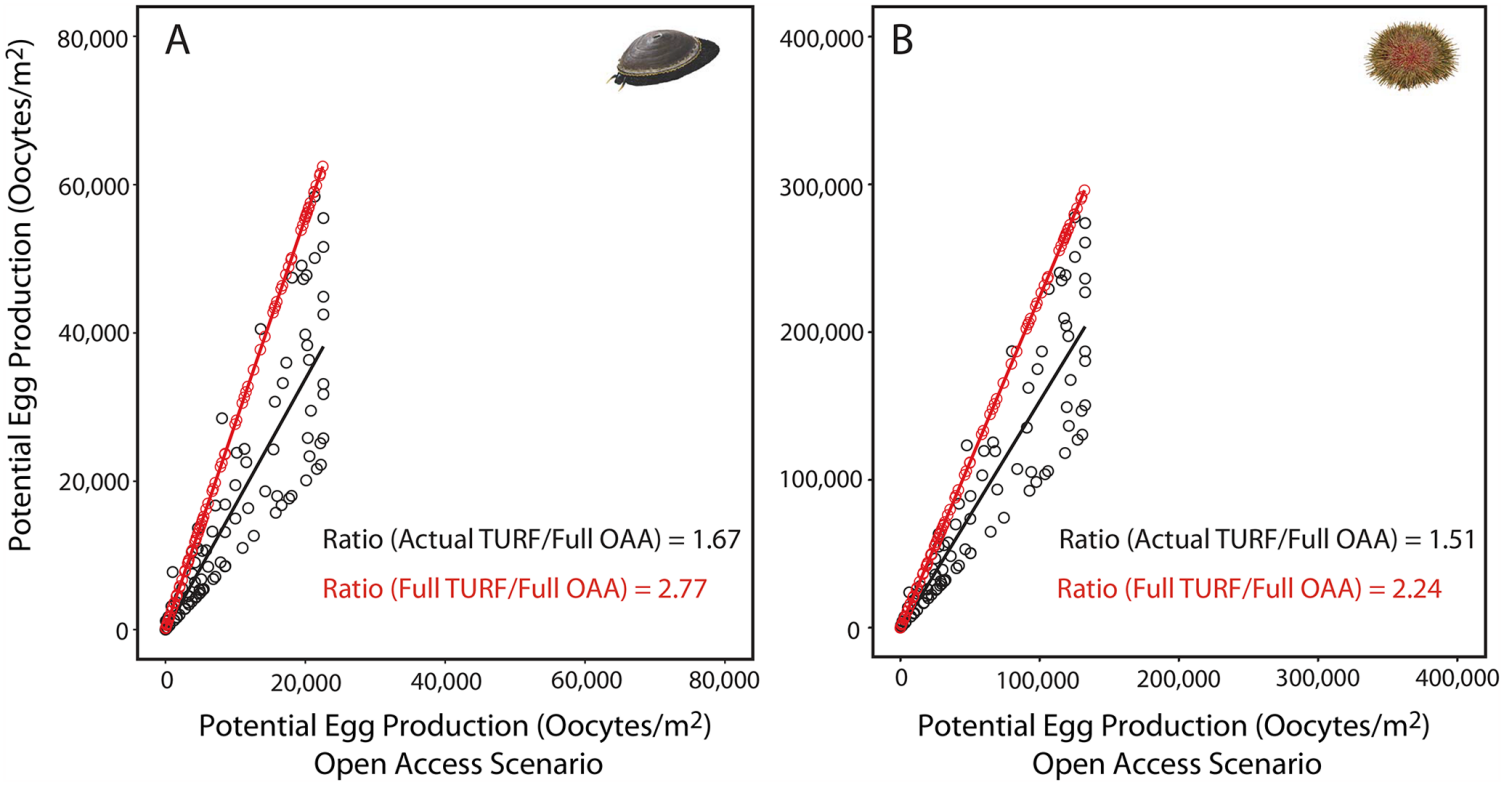
Influence of fishing on potential egg production. Comparison of potential egg production between the open access areas (OAAs) scenario against (a) current scenario (existing Territorial Use Rights for Fisheries, TURFs; black line and symbols) and (b) full protection (all TURFs; red line and symbols) for (A) *Fissurella latimarginata* and (B) *Loxechinus albus*. The numbers in the lower right corner in each plot show the ratio in potential egg production from the two scenarios.

The spatial distribution of potential egg production predicted by the model showed similar patterns for both species, driven by the distribution of suitable habitat. Suitable habitat explained 91.57% (F = 2652.60, df = 1, p<0.001) and 94.78% (F = 2788.02, df = 1, p<0.001) of total variability in potential egg production of keyhole limpets and sea urchins, respectively. The highest percentage of potential egg production occurred in the north region of studied domain, where about 80% of potential egg production of both species were concentrated between 31.57°S and 33.50°S (Fig 3). In the southern region of the studied domain (33.50°S to 36.00°S), where suitable rocky habitat is present only along 24% of the coastline, egg production was lower. However, fishing regime influenced potential egg production in suitable habitats. The system of TURFs in our study region increased potential egg production of *F*. *latimarginata* by 67% against the predicted production under a complete OAAs scenario (Fig 4A). The increase was smaller for *L*. *albus* (52%; Fig 4B). Our predictions also showed the enormous influence of fishing in reducing potential egg production of both species. Potential egg production showed more than a twofold reduction when the complete TURFs scenario was compared against complete OAAs condition in both species (Fig 4A and 4B).

Size explained a similar proportion of the variability in potential egg production in both species (keyhole limpet: 23.09%, sea urchin: 20.22%; Table 3). However, the contribution of density to explain the variability in egg production potential was different between both species. Density explained 37.26% of the total variability in potential egg production in keyhole limpets and 79.77% in sea urchins (Table 3).

**Table 3:** Influence of size and density on potential egg production. Statistical results of the effect of density and size on potential egg production.

**A.** *Fissurella latimarginata*					
Model: ANCOVA (Potential Egg Production ~ Density + Size)					
	Df	Sum Sq	Mean Sq	Pr (>| F |)	Exp Var (%)
Density	1	6.62*10^8^	6.62*10^8^	0.08	37.26
Size	1	4.10*10^8^	4.10*10^8^	0.15	23.09
Residuals	5	7.04*10^8^	1.42*10^8^		
**B. *Loxechinus albus***					
Model: ANCOVA (Potential Egg Production ~ Density + Size)					
	Df	Sum Sq	Mean Sq	Pr (>| F |)	Exp Var (%)
Density	1	1.86*10^10^	1.86*10^10^	1.47*10^−3^	79.77
Size	1	4.72*10^9^	4.72*10^9^	2.92*10^−3^	20.22
Residuals	1	9.92*10^4^	9.92*10^4^		

## Discussion

In a global context, our main results show the influences of TURFs in enhancing size and density of exploited species and the direct consequences of enhanced size and density on potential egg production, thereby suggesting an important role of partially protected areas in producing propagules to seed exploited areas. Our results show further influences of management regime, as potential egg production was up to 200% lower in open access areas. Finally, although we did not find consistent results for both species we were able to determine enhanced potential egg production. Locally, our results are relevant for identifying the main regions along the coast of central Chile that exhibit higher potential for egg production in two important, exploited resources, the keyhole limpet (*F*. *latimarginata*) and the red sea urchin (*L*. *albus*).

In accordance with published evidence, our results show the general benefits of protection enhancing relevant biological variables, such as abundance and/or adult size inside TURFs boundaries [20,21]. Although fully protected areas offer greater benefits than partially protected areas (74% greater density and 30% larger sizes, [21]), partially protected areas, such as TURFs, also generally showed significant effects in these two biological variables. Our results comparing TURFs and OAAs show similar patterns in density and size of exploited resources as expected from a synthesis of empirical studies comparing these biological variables in partially protected and background areas across a range of geographic locations worldwide (on average, 36% higher density, and 10% larger sizes in protected areas than in background areas [21]). We found that sizes were between 2% and 13% larger, and density between 65% and 238% higher (although non-significant for sea urchin) in TURFs than in open access areas. The large variability in density of sea urchins both in TURFs and open access areas may have precluded us from identifying a clear and significant pattern of higher density in TURFs for both species. Previous studies have also reported a lower impact of protection on *L*. *albus*, and larger variability in abundance, probably related to the patchy distribution of suitable microhabitats [20]. *L*. *albus* is predominantly associated with highly wave exposed zones, where they are frequently found forming aggregations.

Our results also suggest that the enhanced biological variables can potentially amplify the benefits of protection outside the boundaries of protected areas by increasing individual production of dispersive propagules [17–19]. First, the models we used to analyze potential fecundity showed an exponential increase in fecundity with size in both species. Second, the 13% differences in keyhole limpets’ size in TURFs compared to OAAs produced a 68% greater potential fecundity of females. The size increase between TURFs and OAA is smaller than the average 30% increase in size reported in a worldwide analysis comparing individual size between no-take and open access areas [18]. Since small changes in size can have tremendous impact on potential fecundity, even the contribution of partially protected areas (10% larger sizes) to potential egg production through size enhancement of reproductive individuals might be substantial [18]. Worldwide evidence shows that on average, the greater sizes in no-take areas, up to 200% larger than in partially protected areas [18], can disproportionally influence dependent variables such as potential egg production. Therefore, it is important to further explore the relevance of enhanced size on potential fecundity in fully and partially protected areas. We recognize that effective larval spillover requires a broader approach, including other factors not considered in this study, from fertilization success to processes affecting planktonic larval phases and local adaptation of early recruits [46,47]. However, enhanced fertilization success can also be predicted in areas of higher population density [48] such as TURFs or no-take areas. It is also important to keep in mind the variability among species. Our analysis also suggests that differences in mean size of 2% between TURFs and OAAs of sea urchins do not produce significant differences in average potential fecundity.

The relevance of density in determining gonad production per unit of area has been clearly shown in the literature [32–34]. However, the interplay between the combined effects of density and size is less clear and our results did not yield clear conclusions in this direction. In the case of keyhole limpets, for which density and size were enhanced in TURFs areas, a significant difference in potential egg production was recorded. The comparison between the two species showed that potential egg production of *F*. *latimarginata* increased by protection 277% more than *L*. *albus* due to the combined influence of larger size and higher densities in TURFs. In spite of the fact that we did not find significant differences in sea urchin density and size associated to fishing regime, the spatial variability in potential egg production of sea urchin is mainly explained by density (80%) and secondly by size (20%). Size differences ranging between 2 and 13% between open access and TURFs areas seem to explain about 20% of the variability in potential egg production. Thus, our results suggest that density might have a larger influence on variability in potential egg production than individual size. However, a third factor to consider is the direct influence of protection on reproductive investment, and therefore egg production. So far, most evidence is insufficient for determining the relative importance of protection indirectly on adult size and density, and directly on gonad investment in enhancing reproductive potential in protected areas. Direct estimates of gonad weight in scallops (*Pecten maximus*) suggest that there can be an additional benefit of protection directly on gonad investment, with differences of gonad weight ranging between 20 and 25% in protected areas [36]. Therefore, it is important to increase the available information in general, to assess the direct influence of protection on gonad production. Further explorations, however, are needed to understand the most relevant determinants of egg production in protected areas, and to advance in strategies to enhance the benefits of protection outside the boundaries of protected areas (e.g., placing protected areas in sites assuring spill over, based on circulation patterns).

Our results show that the system of TURFs areas in our study region enhanced potential egg production of *F*. *latimarginata* by 67% in comparison to a complete open access scenario. In the case of sea urchins, a 52% greater potential egg production was estimated. Although a similar trend towards increasing potential egg production has been observed in other invertebrate species [31,33,34], the magnitude of the change is substantially different. Empirical evidence predicts between 2 to 5 times greater egg production in pink abalone (*Haliotis corrugata*) [31]. A 10% increase in egg production of lobster (*Jasus edwarsii*) per year after closure was estimated, suggesting a 4 fold increase over 15 years (average age of the partially protected areas studied here). We relate the smaller changes in the two species studied here to the fact that the protected areas used in this study are also fishing areas (partially protected). Consistently, the comparison of our results of egg production against studies conducted with invertebrates in no-take areas in other regions show that fully protected areas can provide higher benefits than partially protected areas, such as TURFs. However, partial protection may be the only possible form of protection in highly populated regions [38], and can still have significant positive influence in potential seeding. In our case, the influence of fishing is so high, that a 277% greater potential egg production of *F*. *latimarginata* can be expected between a complete OAAs scenario in comparison with a TURFs scenario. This hypothetical comparison against a no-take scenario is more similar to the studies comparing no-take areas in other invertebrate species [31,33,34]. In the case of *L*. *albus* a 224% increase can be expected. In our study region, we did not have enough no-take areas to include a set to compare the three levels of human impact (fishing regime) to really evaluate the influence of a gradient in fishing effort on potential egg production. However, our results suggest that the intermediate effect of TURFs can still contribute substantially to enhancing egg production in the study area and potentially contribute to seeding exploited areas.

Our results also provide insights for management and conservation at the local level. The highest percentage of potential egg production was estimated in the north region of studied domain for both species, following the distribution of suitable habitat. More than 80% of potential egg production of *F*. *latimarginata* and *L*. *albus* were produced between 31.57°S and 33.50°S. It is worth noticing that the northern part of our study area also concentrates more than 90% of landings of both species (SERNAPESCA 2013). This pattern may be driven not only by abundance of resources (and habitat), but also by differences in fishing pressure among sites [49]. The map of potential egg production, combined with other processes, such as coastal circulation patterns, can be a key element to design a network of partially and fully protected areas in one of the most productive, yet exploited coastal regions of the world. Our results show the relevance of focusing conservation efforts in the most important area for potential egg production, the northern domain of our study area, although it may imply restricting fishing effort. However, the potential distribution of propagules needs to be coupled with hydrodynamic and biological models would allow to improve our understanding of larval supply to different areas of the coast, by identifying source and sink populations [50].

## Supporting information

S1 Table Reproductive parameters of keyhole limpet and urchin used as fixed parameters in the potential egg production equation, (Eqs 2 and 3). S indicates proportion of mature individuals and K proportion of *F*. *latimarginata*.
(DOCX)

S2 Table Keyhole limpet sex ratio data. (CSV)

S3 Table Urchin sex ratio data. (CSV)

S4 Table Data of density of individuals per transect. (CSV)

S5 Table Keyhole limpet potential fecundity data. (CSV)

S6 Table Urchin potential fecundity data. (CSV)

S7 Table Spatial information on a 2-km latitudinal grid. (CSV)

S8 Table Keyhole limpet model result. (CSV)

S9 Table Urchin model result. (CSV)

S10 Table Raw data information. (CSV)
