# Supplementary material for: Egg production patterns of two invertebrate species in rocky subtidal areas under different fishing regimes along the coast of central Chile
Source: PLoS One. 2017 May 8;12(5):e0176758. doi: 10.1371/journal.pone.0176758 (PMC5421777; doi:10.1371/journal.pone.0176758)
Supplement: S1 Table — S indicates proportion of mature individuals and K proportion of F. latimarginata. (DOCX) [file pone.0176758.s001.docx]

| ***Fissurella latimarginata*** | | | | | | | | ***Loxechinus albus*** | | | | | | |
| --- | --- | --- | --- | --- | --- | --- | --- | --- | --- | --- | --- | --- | --- | --- |
| **Name** | **Sampling year** | **S** | | | **K** | | | **Name** | **Sampling**  **year** | **S** | | | | |
|  |  | Total  sampled | Mature | (%) Mature | Total  sampled | Num.  *(F latimarginata)* | %  *(F. latimarginata)* |  |  | Total  sampled | | Mature | (%) Mature | |
| Chigualoco | 2006 | 196 | 143 | 0.73 | 1449 | 196 | 0.14 | Bonifacio sector A | 2008 | 41 | | 24 | 0.59 | |
| Chigualoco | 2007 | 220 | 193 | 0.88 | 537 | 220 | 0.41 | Bonifacio sector B | 2007 | 103 | | 79 | 0.77 | |
| El Quisco sector A | 1999 | 372 | 217 | 0.58 | 756 | 372 | 0.49 | Bonifacio sector B | 2009 | 227 | | 201 | 0.89 | |
| El Quisco sector A | 2000 | 78 | 77 | 0.99 | 225 | 78 | 0.35 | Chaihuin sector C | 2003 | 42 | | 32 | 0.76 | |
| El Quisco sector A | 2003 | 45 | 45 | 1.00 | 236 | 45 | 0.19 | Chan chan | 2009 | 162 | | 114 | 0.70 | |
| El Quisco sector A | 2004 | 95 | 94 | 0.99 | 284 | 95 | 0.33 | El Quisco sector A | 2005 | 382 | | 361 | 0.95 | |
| El Quisco sector A | 2005 | 167 | 166 | 0.99 | 744 | 167 | 0.22 | El Quisco sector A | 2006 | 174 | | 174 | 1.00 | |
| El Quisco sector A | 2006 | 162 | 160 | 0.99 | 705 | 162 | 0.23 | Farellones de Carelmapu | 2003 | 193 | | 119 | 0.62 | |
| El Quisco sector A | 2009 | 117 | 116 | 0.99 | 713 | 117 | 0.16 | Farellones de Carelmapu | 2004 | 258 | | 132 | 0.51 | |
| Huentelauquen | 2006 | 33 | 29 | 0.88 | 440 | 33 | 0.08 | Farellones de Carelmapu | 2005 | 214 | | 139 | 0.65 | |
| Laguna Verde sector C | 2004 | 542 | 495 | 0.91 | 843 | 542 | 0.64 | Farellones de Carelmapu | 2007 | 184 | | 77 | 0.42 | |
| Laguna Verde sector C | 2005 | 298 | 275 | 0.92 | 669 | 298 | 0.45 | Farellones de Carelmapu | 2008 | 201 | | 147 | 0.73 | |
| Laguna Verde sector C | 2007 | 279 | 262 | 0.94 | 536 | 279 | 0.52 | Horcon | 2001 | 212 | | 162 | 0.76 | |
| Los Vilos sector B | 2000 | 489 | 428 | 0.88 | 978 | 489 | 0.50 | Horcon | 2002 | 680 | | 608 | 0.89 | |
| Los Vilos sector B | 2001 | 580 | 356 | 0.61 | 672 | 580 | 0.86 | Horcon | 2003 | 281 | | 213 | 0.76 | |
| Los Vilos sector B | 2002 | 333 | 271 | 0.81 | 690 | 333 | 0.48 | Isla doña Sebastiana | 2004 | 121 | | 44 | 0.36 | |
| Los Vilos sector B | 2003 | 428 | 314 | 0.73 | 751 | 428 | 0.57 | Isla doña Sebastiana | 2005 | 388 | | 188 | 0.48 | |
| Los Vilos sector B | 2004 | 607 | 518 | 0.85 | 1107 | 607 | 0.55 | Isla doña Sebastiana | 2006 | 756 | | 381 | 0.50 | |
| Los Vilos sector B | 2006 | 447 | 301 | 0.67 | 767 | 447 | 0.58 | Isla doña Sebastiana | 2007 | 453 | | 153 | 0.34 | |
| Los Vilos sector B | 2007 | 282 | 249 | 0.88 | 504 | 282 | 0.56 | Isla doña Sebastiana | 2008 | 440 | | 293 | 0.67 | |
| Los Vilos sector B | 2008 | 287 | 240 | 0.84 | 470 | 287 | 0.69 | La Cachina | 2008 | 299 | | 216 | 0.72 | |
| Los Vilos sector B | 2009 | 257 | 191 | 0.74 | 373 | 257 | 0.69 | Laguna Verde sector C | 2004 | 189 | | 144 | 0.76 | |
| Los Vilos sector C | 2003 | 143 | 74 | 0.52 | 574 | 143 | 0.25 | Laguna Verde sector C | 2005 | 486 | | 298 | 0.61 | |
| Los Vilos sector C | 2004 | 515 | 460 | 0.89 | 1193 | 515 | 0.43 | Laguna Verde sector C | 2007 | 301 | | 232 | 0.77 | |
| Los Vilos sector C | 2006 | 229 | 193 | 0.84 | 770 | 229 | 0.29 | Los molinos sector A | 2002 | 31 | | 25 | 0.77 | |
| Mississipi | 2002 | 348 | 289 | 0.83 | 457 | 348 | 0.76 | Los Vilos sector B | 2001 | 78 | | 60 | 0.77 | |
| Papudo | 2003 | 479 | 381 | 0.79 | 702 | 479 | 0.68 | Los Vilos sector B | 2002 | 251 | | 135 | 0.34 | |
| Papudo | 2004 | 348 | 289 | 0.83 | 848 | 348 | 0.41 | Los Vilos sector B | 2008 | 247 | | 247 | 1.00 | |
| Papudo | 2005 | 617 | 505 | 0.82 | 909 | 617 | 0.68 | Los Vilos sector C | 2004 | 492 | | 263 | 0.53 | |
| Pichidangui | 2006 | 26 | 23 | 0.88 | 288 | 26 | 0.09 | Mehuin sector B | 2003 | 637 | | 539 | 0.85 | |
| Totoralillo Sur | 2007 | 145 | 126 | 0.87 | 615 | 145 | 0.24 | Mehuin sector B | 2005 | 282 | | 240 | 0.85 | |
| Totoralillo sur-Las Plaillas | 2007 | 81 | 68 | 0.84 | 532 | 81 | 0.15 | Mehuin sector B | 2006 | 455 | | 447 | 0.98 | |
| Mean |  |  |  | **0.84** |  |  | **0.43** | Mean |  |  |  | | | **0.69** |

**S1 Table. Reproductive parameters of keyhole limpet and urchin used as fixed parameters in the potential egg production equation, (Eqs. 2 and 3).** S indicates proportion of mature individuals and K proportion of *F. latimarginata.*
